# Data on the phosphorylation state of the catalytic serine of enzymes in the α-D-phosphohexomutase superfamily

**DOI:** 10.1016/j.dib.2016.12.017

**Published:** 2016-12-15

**Authors:** Yingying Lee, Cristina Furdui, Lesa J. Beamer

**Affiliations:** a Departments of Biochemistry and Chemistry, University of Missouri-Columbia, Columbia, MO 65211, United States; b Department of Internal Medicine, Wake Forest University Health Sciences, Winston-Salem, NC, USA

**Keywords:** Phosphorylation, Enzymes, Mass spectrometry, Active site

## Abstract

Most enzymes in the α-D-phosphohexomutase superfamily catalyze the reversible conversion of 1- to 6-phosphosugars. They play important roles in carbohydrate and sugar nucleotide metabolism, and participate in the biosynthesis of polysaccharides, glycolipids, and other exoproducts. Mutations in genes encoding these enzymes are associated with inherited metabolic diseases in humans, including glycogen storage disease and congenital disorders of glycosylation. Enzymes in the superfamily share a highly conserved active site serine that participates in the multi-step phosphoryl transfer reaction. Here we provide data on the effects of various phosphosugar ligands on the phosphorylation of this serine, as monitored by electrospray ionization mass spectrometry (ESI-MS) data on the intact proteins. We also show data on the longevity of the phospho-enzyme under various solution conditions in one member of the superfamily from *Pseudomonas aeruginosa*, and present inhibition data for several ligands. These data should be useful for the production of homogeneous samples of phosphorylated or unphosphorylated proteins, which are essential for biophysical characterization of these enzymes.

**Specifications Table**

**Table:** 

Subject area	*Chemistry, Biology*
More specific subject area	*Biochemistry, Enzymology*
Type of data	*Tables, figures*
How data was acquired	*Mass spectrometry using a NanoLC-Nanospray QTOF (Agilent 6520) with C8 column chromatograph; enzyme inhibition assays*
Data format	*Analyzed*
Experimental factors	*Purified proteins were incubated with phosphosugar ligands and under different solution conditions; enzyme inhibition was assessed in presence of ligands*
Experimental features	*ESI-MS data on intact proteins were collected and the % phosphorylation of the catalytic serine calculated; enzyme activity was measured on a spectrophotometer using a colorimetric assay*
Data source location	*University of Missouri Charles W. Gehrke Proteomics*
	*Center, Columbia, MO USA*
Data accessibility	*The data is available within this article*

**Value of the data**

• The catalytic phosphoserine of enzymes in the α-D-phosphohexomutase superfamily plays an integral role in enzyme mechanism.
• We present data on the phosphorylation state of the serine under varying conditions.
• These data will be useful information for preparation of homogeneous samples of phospho- or dephospho-enzyme for future biophysical and kinetic studies.

## 1 Data

The data in this article show the effects of phosphosugar ligands and other variables on the phosphorylation state of the catalytic serine in several enzymes in the α-D-phosphohexomutase superfamily. Data were obtained by ESI-MS. Data on enzyme inhibition by several ligands is also presented.

## 2 Experimental design, materials and methods

### 2.1 Materials

*Leuconostoc mesenteroides* glucose 6-phosphate dehydrogenase (G6PDH), and all bis- and monophosphorylated sugars except xylose 1-phosphate (X1P) were obtained from Sigma-Aldrich. X1P was kindly synthesized by Dr. Thomas Mawhinney (University of Missouri).

### 2.2 Preparation of protein samples

Expression and purification of *Pseduomonas aeruginosa* PMM/PGM (PaPMM), *Bacillus anthracis* phosphoglucosamine mutase (BaPNGM), *Salmonella typhimurium* PGM (StPGM), *Francisella tularensis* PNGM (FtPNGM), and human phosphoglucosmutase 1 (hPGM1) were performed as described previously [1], [2], [3], [4]. Purified proteins were dialyzed into 50 mM MOPS, pH 7.4, concentrated, and stored at −80 °C until use.

### 2.3 Incubation with phosphosugar ligands

Ligands tested for effects on phosphorylation included two bisphospho-sugars, and various monophosphosugars (e.g., substrate or product in the enzyme reaction), which have been reported, in different instances, to either phosphorylate or dephosphorylate these enzymes [5], [6]. The compounds were prepared as aqueous stock solutions at 1–200 mM, and mixed with protein to determine their effect on the phosphorylation level of the active site serine. For mass spectrometry, enzymes at 40–120 M were incubated with a 6.25 M excess of compound for 18 h at 4 °C. Samples were flash frozen and stored at −80 °C until analysis.

### 2.4 ESI-MS data collection and analysis

Analysis of intact proteins by mass spectrometry was done as described previously [7] with a NanoLC-Nanospray QTOF (Agilent 6520) and C8 column chromatography. Expected and observed molecular masses of the proteins are found on Table 1. Duplicate spectra of two identical samples showed phosphorylation levels within 2% of each other, indicating good reproducibility. No degradation of the protein samples was observed during any of the conditions tested.

The percentage phosphorylation was calculated by normalizing the sum of the dephosphorylated and phosphorylated peak heights to 1.0. As the proteins characterized herein are known to be phosphorylated on the conserved active site serine, and ESI-MS data confirmed a single phosphorylation site, no additional attempts were made to localize the site of phosphorylation. The exception to this was StPGM, which showed two phosphorylation sites via ESI-MS (see Supplementary Methods and Fig. S1).

### 2.5 Enzyme inhibition assays

Enzymatic activity of PaPMM was quantified by measuring the formation of glucose 6-phosphate (G6P) in a coupled assay with G6PDH. The conversion of NAD to NADH was monitored by UV–vis spectrophotometry on a CARY 100 spectrophotometer at 25 °C, as previously described [3]. Time courses of enzyme activity in the presence of glucosamine 1-phosphate (GlcN1P) and glucosamine 6-phosphate (GlcN6P) were conducted using 0.14 µM enzyme with 0.5 µM of glucose 1,6-bisphosphate (G16P), and 135 µM of substrate, glucose 1-phosphate (G1P).

*Ki* values for GlcN1P and the substrate analog X1P were determined as follows.For X1P, assays were performed with 0.1 µM enzyme, 1 µM G16P, and 10–500 µM G1P (substrate). For GlcN1P, assays were performed with 0.3 µM enzyme, 0.5 µM G16P, and 6.8–272 µM G1P. Data were fitted to the Michaelis–Menten equation. Apparent *K*_*m*_ (for X1 P studies) or apparent *k*_*cat*_ (for GlcN1P) values obtained at each inhibitor concentration were fitted using Eq. (1) or (2), respectively, to calculate *K*_*I*_, the inhibitor dissociation constant. In Eq. (1) and (2), I is the concentration of inhibitor, *K*_*m*_ and *k*_*cat*_ are the Michaelis parameters of enzyme without the inhibitor.

(1) $$K_{m,app}=K_{m}\;x\left(1+\frac{\left[\;I\;\right]}{\left[K_{I}\right]}\right)\;\mathrm{Competitive}\;\mathrm{Inhibition}$$

(2) $$k_{cat,app}=k_{cat}\;/\;\left(1+\frac{\left[\;I\;\right]}{\left[K_{I}\right]}\right)\;\mathrm{Noncompetitive}\;\mathrm{Inhibition}$$

### 2.6 Data on phosphorylation by bisphosphosugars

**Fig. 1:**
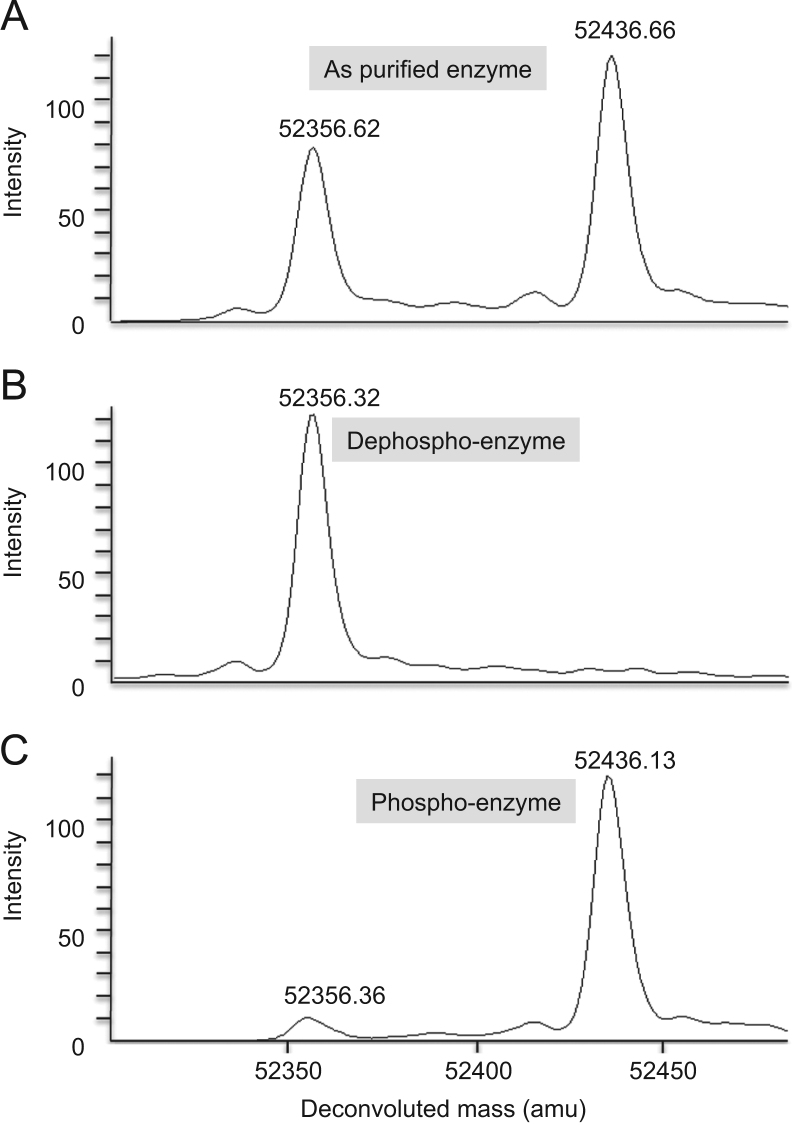
ESI-MS spectra of various forms of PaPMM. Protein samples shown are: (A) as purified; (B) unphosphorylated; and (C) phosphorylated. Intensity is on an arbitrary scale. Peak at 52,356 corresponds to unphosphorylated protein; peak at 52,436 corresponds to protein phosphorylated at residue 108.

A broad assessment of the effects of phosphosugar ligands on the phosphorylation of the catalytic serine was conducted on PaPMM, one of the best-characterized members of the superfamily [8], [9], [10], [11]. Phospho- and dephospho-enzyme are easily distinguished in spectra (Fig. 1). Data on the effects of ligands are shown in Table 2. The phosphorylation level can be increased to ~95% through incubation with G16P, a known activator for the superfamily. An alternate bisphosphosugar, fructose 1,6-bisphosphate (F16P), also increases the phosphorylation level of PaPMM to ~85%.

Several other sequence-diverse proteins in the superfamily were examined for changes in phosphorylation: BaPNGM, FtPNGM, StPGM, and hPGM1. For all proteins, both G16P and F16P increase phosphorylation level of the active serine (Table 3). For two of the proteins, FtPNGM and StPGM, F16P appears to be somewhat more effective than G16P under the conditions tested, with phosphorylation levels after incubation ranging from 90–100%.

As noted in Section 2.4, ESI-MS of StPGM indicated two phosphorylation sites. To determine if one of these was the catalytic phosphoserine, the protein was subjected to proteolysis and phosphopeptides identified (Supplemental methods). A single phosphopeptide was identified by these studies (Fig. S1), corresponding to residues 134–156, which includes the active site serine (residue 146). Only one region of the protein was not covered by this analysis (residues 42–53), which could contain the second site of phosphorylation as it includes two serines and one threonine.

### 2.7 Data on dephosphorylation of enzymes

Incubations of PaPMM with G1P and G6P and the related sugars GlcN1P and GlcN6P were performed (Table 1). Under the conditions tested, most of the monophosphosugars were associated with a reduction in the level of phosphorylation, by up to 50%. Incubation with GlcN1P, however, substantially reduces the phosphorylation level of PMM/PGM, by ~90%, consistent with previous observations [12]. GlcN1P was also found to reduce phosphorylation of hPGM1, but had no effect on StPGM or PNGM proteins (Table 3).

**Fig. 2:**
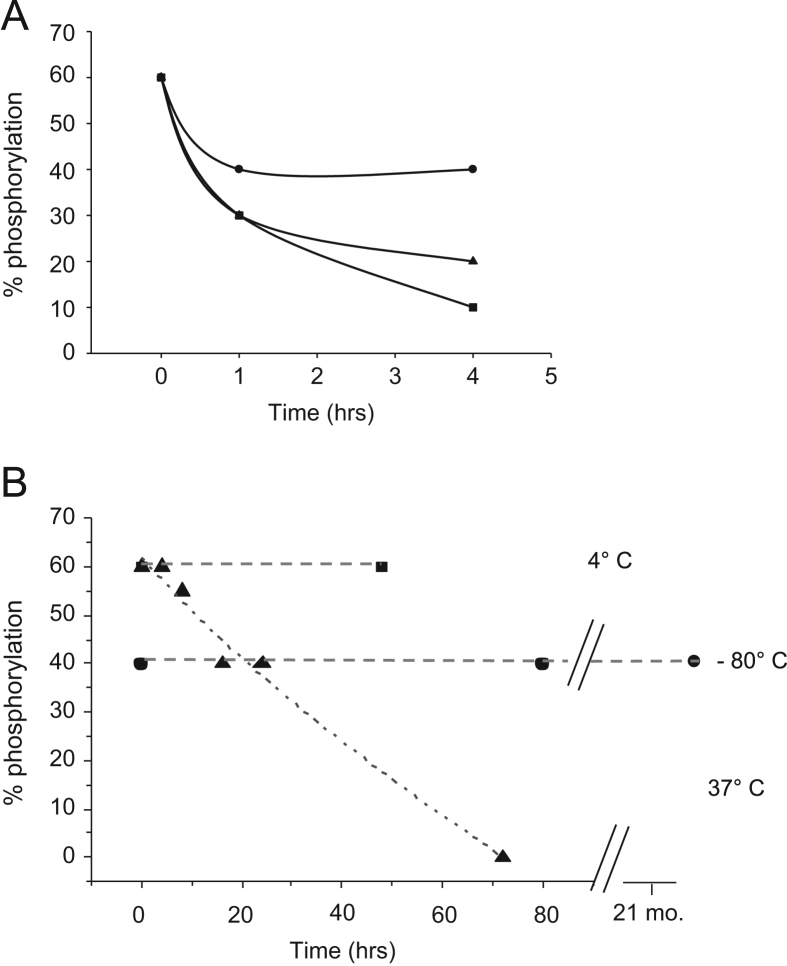
Time courses of dephosphorylation of PaPMM. (A) Concentration and time dependence of dephosphorylation in the presence of GlcN1P at 4 °C: (circles)1x GlcN1P; (triangles) 2x GlcN1P; and (squares) 4x GlcN1P. (B) Time course of dephosphorylation at varying temperatures: (triangles)37 °C; (squares) 4 °C; and (circles) −80 °C. Data were fit using either linear regression or exponential (37 °C) equations.

Several concentrations of G1cN1P were tested at various time points in incubations with PaPMM. A time course of dephosphorylation (i.e., loss of phosphoryl group due to hydrolysis) in the presence of increasing molar equivalents of GlcN1P is shown in (Fig. 2A). The effect of temperature on the longevity of phosphorylation of PMM/PGM was also assessed. Samples were collected after incubation of the protein at 4° and 37 °C for various length of times, and after prolonged storage at −80 °C. The phosphorylation level was unchanged at 4 °C for at least two days and at −80 °C for 21 months. At 37 °C, a reduction in % phosphorylation was observed within 8 h, with complete loss over three days (Fig. 2B).

### 2.8 Data on the effects of ligands on enzyme activity

**Fig. 3:**
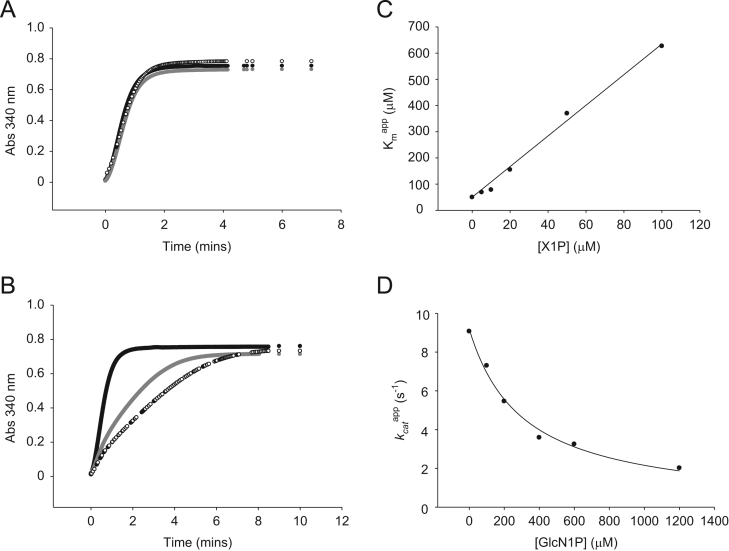
Time course profiles and inhibition profiles of PaPMM activity. (A) Activity time course with GlcN6P concentrations of 0 μM (circles), 200 μM (gray circles) and 500 μM (open circles). (B) Activity time course with GlcN1P concentrations of 0 μM (circles), 267 μM (gray circles), and 300 μM (open circles). Plots showing the inhibition of PaPMM by (C) the substrate analog X1P and (D) GlcN1P. Kinetic parameters for X1P derived using Eq. (1) are: *k*_*cat*_ = 13±1 s^−1^, *K*_*i*_ = 8.6±0.3 mM, *K*_*m*_ 52±9 mM. Kinetic parameters for GlcN1P derived using Eq. (2) are: *k*_*cat*_ 9.2±0.2 s^−1^; *K*_*i*_ 307±29 μM; *K*_*m*_ 28±2.8 μM. Unlike X1P, which is a competitive inhibitor, GlcN1P displays a noncompetitive inhibition pattern.

To assess interactions between PaPMM and G1cN1P or GlcN6P, these two phosphosugars were tested as inhibitors for the PGM activity (Fig. 3). GlcN6P shows no effect on time courses of enzyme activity at the concentrations tested (Fig. 3A), while GlcN1P shows increasing reductions in activity at higher concentrations (Fig. 3B). As a control, the substrate analog X1P, which has the same stereochemistry as glucose but lacks the O6 hydroxyl necessary for phosphoryl transfer, was also assessed as an inhibitor. The effects of X1P are consistent with those of a competitive inhibitor (Fig.3C), with a *K*_i_ of 8.6±0.3 μM. GlcN1P shows evidence of noncompetitive inhibition with a *K*_i_ of 307±29 μM (Fig. 3D).

These data provide information on ligands and solution conditions that affect the phosphorylation state and lifetime of the active site serine of enzymes in the α-D-phosphohexomutase superfamily, and may assist with preparation of homogeneous protein samples for further studies.

## Figures and Tables

**Table 1:** Calculated and observed molecular weights by ESI-MS of proteins in this study.

Protein	UniProtKB	Calculated MW^a^		Observed MW	
		Dephospho	Phospho	Dephospho	Phospho
PaPMM	P26276	52355.58	52435.58	52356.62	52436.66
FtPNGM	Q5NII8	50922.58	51002.58	50922.80	51003.06
BaPNGM^b^	Q81VN7	50978.90	51058.90	50979.70	51059.44
StPGM	Q8ZQW9	60827.49	60907.49	60828.97	60905.95
hPGM1	P36871	64115.95	64195.95	64116.10	64196.52

a Based on amino acid sequence of recombinantly expressed proteins, including affinity tags.

b Calculated MW of BaPNGM corrected for an error in the amino acid sequence deposited in the PDB entry (3I3W), which was missing a phenylalanine at the end of the affinity tag.

**Table 2:** Effect of various phosphosugars on phosphorylation of PaPMM.

Phosphosugar	Phosphorylation level (%)	% difference	Relative change
Untreated	60	–	–
Glucose 1,6-bisphosphate	95	+35	↑ 1.6x
Fructose 1,6-bisphosphate	85	+25	↑ 1.4x
Glucose 1-phosphate	30	−30	↓ 2x
Glucose 6-phosphate	30	−30	↓ 2x
Fructose 1-phosphate	50	−10	↓ 1.2x
Fructose 6-phosphate	40	−20	↓ 1.5x
Glucosamine 1-phosphate	6	−54	↓ 10x
Glucosamine 6-phosphate	46	−14	↓ 1.3x

Phosphorylation level estimated from ESI-MS as described in text. Column 1 indicates compound used for incubation. Untreated refers to the protein as purified from recombinant expression system.

**Table 3:** Assessment of phosphorylation level of active site serine of various α-D-phosphohexomutases.

	FtPNGM	BaPNGM^a^	StPGM^b^	hPGM1 c
Untreated	55%	0%	60%	PP
Glucose 1,6-bisphosphate	80%	97%	75%	PP
Fructose 1,6-bisphosphate	100%	90%	90%	92%
Glucosamine 1-phosphate	–	–	–	PP

Dash (−) indicates no observed change in phosphorylation.

a BaPNGM samples were unphosphorylated upon purification, so a decrease in phosphorylation would not be apparent.

b Phosphorylation level estimates for StPGM exclude apparent second phosphorylation site (see text).

c Treatment of hPGM1 with F16P was done herein; data for other incubations were previously published (PP) using similar conditions [4].

## Footnotes

Transparency document Transparency data associated with this article can be found in the online version at: 10.1016/j.dib.2016.12.017.

Appendix A Supplementary data associated with this article can be found in the online version at 10.1016/j.dib.2016.12.017.
