# Supplementary material for: Data on the phosphorylation state of the catalytic serine of enzymes in the α-D-phosphohexomutase superfamily
Source: Data Brief. 2016 Dec 15;10:398–405. doi: 10.1016/j.dib.2016.12.017 (PMC5192239; doi:10.1016/j.dib.2016.12.017)
Supplement: Supplementary file 1 — Supplementary material [file mmc1.docx]

CONFLICT OF INTEREST:

None
