# Supplementary material for: Data on the phosphorylation state of the catalytic serine of enzymes in the α-D-phosphohexomutase superfamily
Source: Data Brief. 2016 Dec 15;10:398–405. doi: 10.1016/j.dib.2016.12.017 (PMC5192239; doi:10.1016/j.dib.2016.12.017)
Supplement: Supplementary file 2 — Supplementary material [file mmc2.pdf]

Supplemental Methods

ESI-MS/MS peptide analysis of StPGM

For the peptide analysis of StPGM, 60 μg of protein was lyophilized and resuspended in 6 M urea, 100 mM HEPES, pH 8.0. An in-solution digest with trypsin was conducted overnight at 37° C. The sample was acidified, lyophilized, and resuspended in 20 μL of 5% acetonitrile and 1% formic acid. A 1 μL aliquot was analyzed on the Agilent QTOF using the phospho-chip (cat # G4240-62021). Two separate runs were conducted to examine non-phosphorylated peptides, followed by examining TiO_2-enriched phospho-peptides.

A search of the NCBInr database (limited to Salmonella sp.) was conducted using Sorcerer2 IDA. Criteria for search included two missed cleavages allowed, carbamidomethy-Cys (fixed), oxidized methionine, and phospho-Ser/Thr/Tyr possible modifications, with 25 ppm mass error allowed. Data were examined using the Scaffold program to determine sequence coverage and validity of peptides identified. Additional manual analysis of the peptide mass data defined regions not identified in the automated search.

Fig. S1

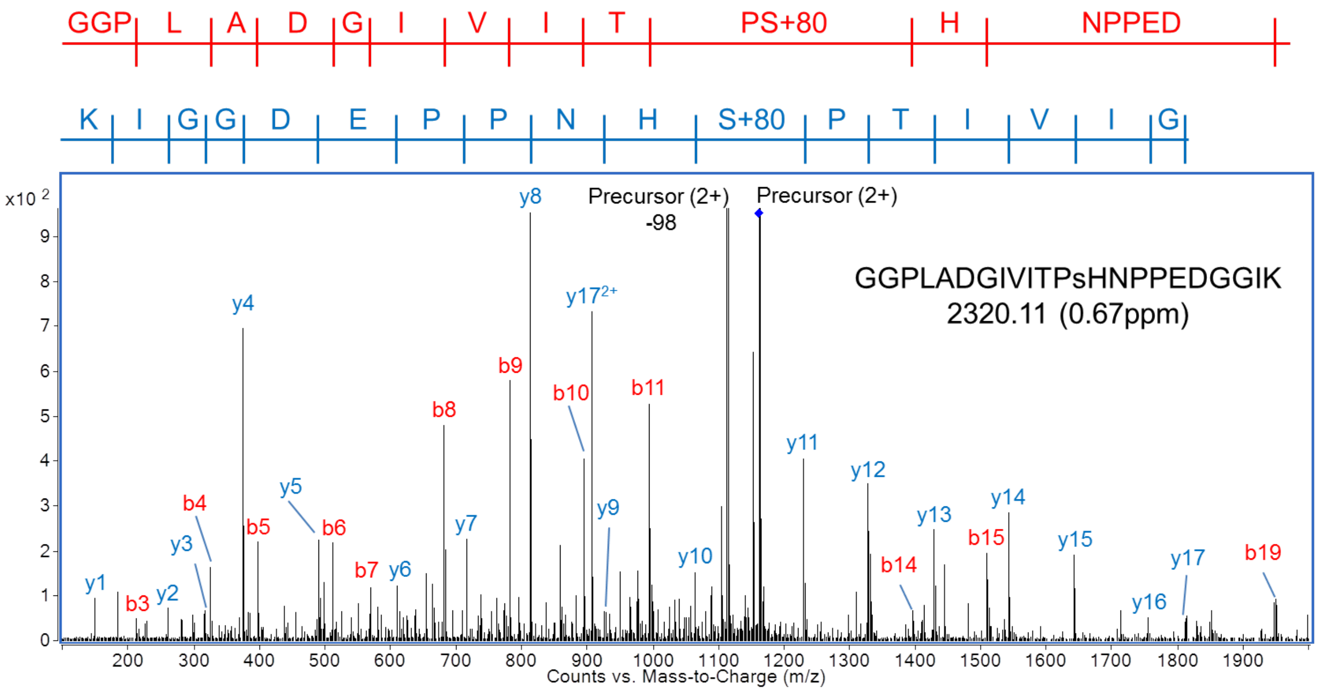

Fig. S1 The MS/MS spectrum for the phosphopeptide containing the active site serine (GGPLADGIVITPSHNPPEDGGIK) in StPGM.
